# Retained Orthodontic Bracket in a Facial Soft Tissue Laceration Following Trauma: Clinical Management and Literature Review

**DOI:** 10.7759/cureus.110027

**Published:** 2026-06-01

**Authors:** R Hariharan, Nithish Kumar, James A Bhagat, G Satheesh, Sakthi S

**Affiliations:** 1 Oral and Maxillofacial Surgery, Adhiparasakthi Dental College and Hospital, Melmaruvathur, IND; 2 Oral and Maxillofacial Surgery, Adhiparasakthi Dental College and Hospital, Melmaruvathurr, IND

**Keywords:** advanced imaging techniques, foreign body management, metalic orthodontic brackets, motor vehicle accident, soft tissue injury

## Abstract

Foreign bodies embedded in the maxillofacial region following traumatic injuries pose a significant challenge to oral and maxillofacial surgeons. These retained objects may become a source of persistent pain, infection, and other complications. Early diagnosis can be achieved through the use of computed tomography (CT), which facilitates accurate localization of the foreign body. Once identified, prompt removal is recommended to prevent further morbidity. Here, we report a case in which early diagnosis of a foreign body embedded within the maxillofacial soft tissues enabled its successful retrieval without complications. Soft tissue impaction of orthodontic brackets following facial trauma is rarely reported in the literature, with only a few isolated case reports describing orthodontic appliances embedded within facial soft tissues after road traffic accidents or sports-related injuries.

## Introduction

Traumatic facial lacerations require careful clinical and radiological assessment because retained foreign bodies may remain undetected during initial wound management and subsequently present with pain, infection, chronic inflammation, delayed healing, or cosmetic deformity. Early identification and localization of embedded foreign materials using computed tomography (CT) are essential for accurate diagnosis and timely surgical retrieval. Previous studies have emphasized that foreign bodies in the facial region are frequently associated with traumatic injuries and may be overlooked during initial evaluation, particularly when deeply embedded within soft tissues [1,2].

Patients undergoing fixed orthodontic treatment represent a unique subgroup in facial trauma, as detached appliance components may become displaced into adjacent soft tissues following impact. Traumatic injuries associated with orthodontic appliances have been reported; however, soft tissue impaction of orthodontic brackets remains uncommon and may be missed during primary wound closure [3,4]. Missing orthodontic components following facial trauma should, therefore, raise suspicion of soft tissue impaction and warrant appropriate radiographic investigation.

Unlike commonly reported retained foreign bodies such as glass, wood, or metallic fragments, the present case involved a displaced orthodontic bracket embedded within the upper labial soft tissue following road traffic trauma. Reports of orthodontic appliance impaction within facial soft tissues are extremely limited, making this case clinically significant because diagnosis was achieved only after suspicion arose from missing orthodontic brackets and subsequent CT evaluation. This case highlights the importance of meticulous wound exploration and radiological evaluation in orthodontic patients presenting with facial trauma.

## Case presentation

A 25-year-old male patient presented to the emergency department (ED) with a history of road traffic accident-related facial trauma. The patient reportedly sustained injuries to the upper lip and adjacent maxillofacial region following the impact. Initial emergency management had been performed at a nearby healthcare facility, where primary wound care and suturing of the facial lacerations were carried out.

The patient sustained facial trauma during a road traffic accident. Primary wound closure was performed at a nearby hospital within two hours of injury. Persistent pain and swelling developed over the subsequent 48 hours, and the patient presented to our institution on the third post-traumatic day. There was no history of loss of consciousness, vomiting, seizures, or bleeding from the ears or nose following the trauma. The patient was conscious, cooperative, and hemodynamically stable upon presentation to the ED. A detailed clinical and radiological assessment was therefore undertaken to evaluate the extent of soft tissue injury and rule out associated facial skeletal trauma or retained foreign bodies.

On clinical presentation, the patient exhibited noticeable facial asymmetry secondary to diffuse soft tissue swelling over the frontal region of the forehead. The swelling was associated with localized tenderness on palpation, suggestive of underlying soft tissue trauma. Multiple superficial abrasions were observed over the forehead and upper lip region, consistent with injuries sustained during the road traffic accident. No active bleeding or discharge was evident at the time of examination. Examination of the temporomandibular joint (TMJ) revealed normal mandibular movements without deviation, restriction, or pain during mouth opening and closing. Extraocular movements were intact in all directions of gaze, and the patient did not report any diplopia, blurring of vision, or other visual disturbances. Neurological assessment showed the patient to be conscious, oriented, and hemodynamically stable, with no evidence of focal neurological deficits (Figure 1).

**Figure 1:**
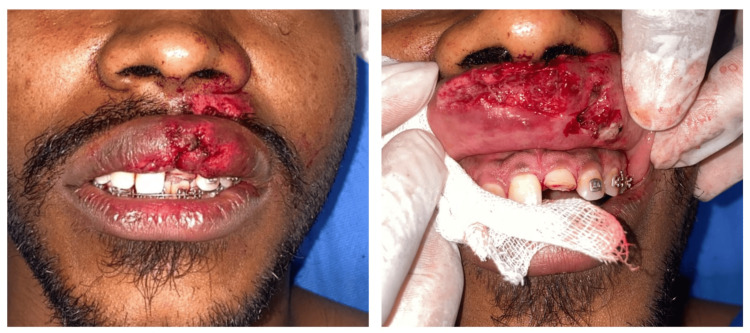
Clinical photographs demonstrating a traumatic laceration and soft tissue injury of the upper lip, with associated intraoral mucosal involvement following facial trauma.

Intraoral examination revealed adequate mouth opening with no evidence of trismus or mandibular deviation. A sutured laceration was noted on the upper labial mucosa, corresponding to the site of previous primary closure performed at the outside healthcare facility. Ellis Class III fractures involving teeth 21 and Ellis Class I fractures involving teeth 31 and 32 were identified clinically. The patient was subsequently referred for restorative management. The patient was undergoing orthodontic treatment and had fixed orthodontic appliances in place, including lower molar bands. On examination, the patient was undergoing fixed orthodontic treatment using pre-adjusted edgewise appliances (0.022-inch MBT prescription). Clinical examination revealed the absence of the orthodontic bracket from the maxillary right central incisor (11,21), corresponding to the region of soft tissue swelling. This finding raised suspicion of traumatic displacement and soft tissue impaction of the bracket.

Based on the clinical findings, persistent swelling and localized tenderness despite previous wound closure, together with the observation of a missing orthodontic bracket, raised suspicion of a retained foreign body. CT imaging was therefore requested to identify any occult embedded foreign material and to exclude associated maxillofacial fractures. After obtaining informed consent, the procedure was carried out under local anesthesia using 2% lignocaine with adrenaline (1:80,000). The wound site was carefully explored following thorough debridement, using 0.9% sodium chloride (normal saline) and 5% povidone-iodine (Betadine) solution to remove debris and reduce the risk of contamination. On meticulous exploration of the lacerated soft tissue, an embedded orthodontic bracket was identified within the upper labial soft tissue and was successfully retrieved. The wound was irrigated thoroughly using 0.9% sodium chloride(normal saline) and managed appropriately following removal of the foreign body (Figure 2).

**Figure 2:**
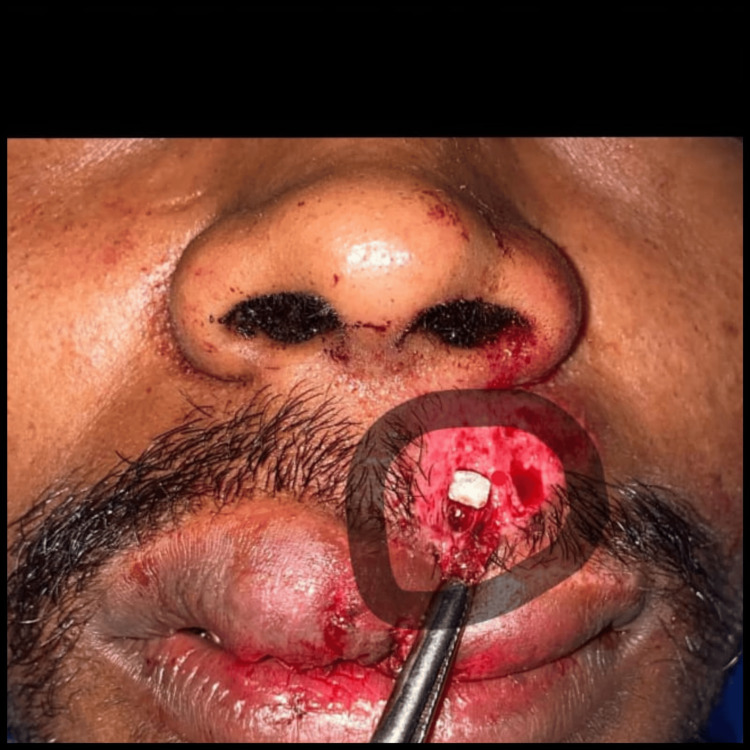
Retrieval of an embedded foreign body from the upper labial region following facial trauma.

Layered wound closure was performed following adequate debridement and retrieval of the foreign body. Closure of the deeper soft tissue layers was achieved using 4-0 Vicryl sutures (Ethicon Inc., Mumbai, India), while skin closure was carried out using 4-0 Ethilon sutures (Ethicon Inc., India) to obtain proper wound approximation and favorable aesthetic healing. Hemostasis was achieved satisfactorily at the end of the procedure.

Postoperatively, the patient was prescribed appropriate medications, including Tab. Cefixime 200 mg twice daily for five days, Tab. Metronidazole 400 mg three times daily for five days, Tab. Paracetamol + Aceclofenac twice daily for five days for analgesia, and Tab. Chymoral Forte twice daily for five days to reduce postoperative inflammation and edema. Dual antibiotic therapy was prescribed because the injury represented a contaminated traumatic wound involving the oral cavity. Cefixime provided coverage against common aerobic organisms, whereas metronidazole offered anaerobic coverage against oral flora potentially introduced during trauma. The patient was also advised regarding maintenance of oral hygiene, wound care precautions, and regular follow-up.

During postoperative follow-up, healing was found to be satisfactory with no evidence of infection, wound dehiscence, persistent swelling, or discharge from the surgical site. At the one-week follow-up visit, the sutures were removed uneventfully, and satisfactory soft tissue healing was observed. On the subsequent second-week follow-up, the wound demonstrated favorable healing with good tissue approximation and acceptable aesthetic outcome. The case emphasizes the importance of meticulous wound exploration, appropriate foreign body retrieval, and proper wound care management in achieving optimal healing and preventing postoperative complications.

Informed written consent was obtained from the patient for the open-access publication of this case report.

Radiological findings

CT imaging was performed approximately 72 hours after injury and 48 hours following primary wound closure. Three-dimensional reconstructed CT imaging and sectional views revealed a radiopaque foreign body embedded within the upper labial soft tissue region, corresponding to a displaced orthodontic bracket. Sagittal and axial CT sections demonstrated the foreign body located superficially within the soft tissue anterior to the maxillary alveolar process without evidence of penetration into adjacent osseous structures. No associated maxillofacial fractures were identified on imaging. The radiographic findings correlated with the clinical suspicion of an impacted orthodontic bracket within the upper lip soft tissue following facial trauma (Figure 3).

**Figure 3:**
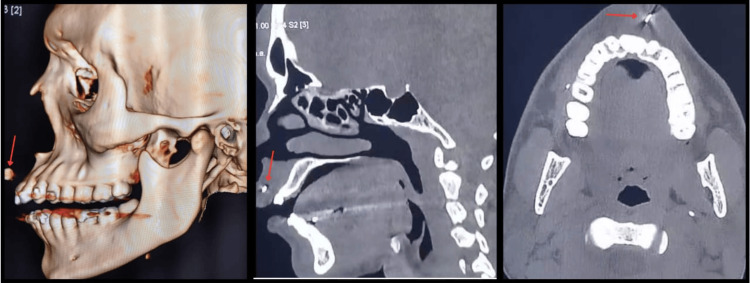
Three-dimensional reconstructed CT image and sagittal and axial CT sections demonstrating a radiopaque foreign body (orthodontic bracket) embedded within the upper labial soft tissues (red arrows).

## Discussion

Retained foreign bodies are a recognized but frequently overlooked complication of facial trauma. Thorough clinical assessment of facial lacerations is essential because embedded foreign materials may remain undetected during initial wound management and subsequently lead to pain, infection, chronic inflammation, delayed healing, or cosmetic deformity. Cho et al. emphasized that meticulous wound exploration and evaluation are critical components in the management of traumatic soft tissue injuries of the face to minimize complications and optimize functional and aesthetic outcomes [1]. Similarly, Rai et al. reported that foreign bodies within the facial region are commonly associated with traumatic injuries and may be missed during the initial assessment, particularly when deeply embedded within soft tissues [2].

The detection of retained foreign bodies relies heavily on appropriate imaging. Clinical examination alone may be insufficient, especially in cases involving soft tissue swelling, previous wound closure, or deeply impacted objects. Abolvardi et al. demonstrated that CT and cone-beam CT (CBCT) are highly effective for identifying foreign bodies within the maxillofacial region and provide valuable information regarding their location and relationship to adjacent structures [5]. Voss et al. further reported that CT imaging offers superior detectability for a wide range of foreign body materials and remains one of the most reliable modalities for localization before surgical retrieval [6]. In the present case, persistent swelling associated with a missing orthodontic bracket prompted CT evaluation, which accurately localized the foreign body within the upper labial soft tissue and facilitated surgical planning. Organic foreign bodies like wood are particularly challenging to detect, as they are often invisible on plain radiographs. Their retention can result in delayed complications such as granulomatous reactions or abscesses. A case series by Pandit et al. (2022) described instances of persistent facial swelling and infection developing months after trauma, ultimately found to be caused by retained wooden splinters detected on MRI [7].

Orthodontic patients represent a unique subgroup in facial trauma because detached appliance components may become displaced into adjacent soft tissues following impact injuries. Samuels et al. highlighted the potential for orthodontic appliances to cause traumatic soft tissue injuries and emphasized the importance of careful evaluation following facial trauma [3]. More recently, Patel et al. reported that missing orthodontic brackets or appliance components should raise suspicion of soft tissue impaction and warrant radiographic investigation to exclude retained foreign bodies [4]. In our patient, the identification of a missing orthodontic bracket during clinical examination was a key finding that raised suspicion of soft tissue impaction and ultimately led to its diagnosis.

The role of antibiotic prophylaxis in maxillofacial trauma remains an area of ongoing discussion. Milic et al., in a systematic review, suggested that prophylactic antibiotics may be beneficial in selected oral and maxillofacial surgical procedures and contaminated traumatic wounds when used alongside appropriate surgical management [8]. In the present case, postoperative antibiotic therapy was prescribed following foreign body retrieval and wound debridement because the injury involved a contaminated facial laceration with oral mucosal involvement. The patient demonstrated satisfactory healing without evidence of infection, wound dehiscence, or persistent inflammation during follow-up.

Psychosocial aspects, including disfigurement and post-traumatic stress disorder (PTSD), are also considerable. Multidisciplinary rehabilitation involving maxillofacial surgeons, psychologists, and physiotherapists is crucial in such complex trauma cases. Surgical outcomes are greatly enhanced when managed under standardized facial trauma protocols and early reconstructive planning [9,10]. Successful management of retained foreign bodies requires timely diagnosis, adequate wound debridement, complete foreign body retrieval, and appropriate wound closure. Failure to remove retained foreign material may result in persistent infection, chronic inflammatory reactions, and delayed wound healing. Following localization of the orthodontic bracket, the wound was explored under local anesthesia, the foreign body was successfully retrieved, and layered closure was performed after thorough irrigation and debridement. These measures contributed to uneventful postoperative healing without infection or wound-related complications.

## Conclusions

We would like to conclude that facial lacerations associated with retained foreign bodies require careful clinical and radiological assessment. In patients undergoing orthodontic treatment, missing brackets or appliance components following trauma should raise suspicion of soft tissue impaction. Early CT evaluation facilitates accurate localization and timely retrieval, thereby preventing infection, chronic inflammation, delayed healing, and unnecessary morbidity. Thorough wound exploration and multidisciplinary management are essential for achieving favorable functional and aesthetic outcomes.
